# Structure and Thermal Expansion of Cu−90 vol. % Graphite Composites

**DOI:** 10.3390/ma14227089

**Published:** 2021-11-22

**Authors:** Andrej Opálek, Štefan Emmer, Roman Čička, Naďa Beronská, Peter Oslanec, Jaroslav Kováčik

**Affiliations:** 1 Institute of Materials and Machine Mechanics, Slovak Academy of Sciences, Dúbravská Cesta 9, 845 13 Bratislava, Slovakia; nada.beronska@savba.sk (N.B.); peter.oslanec@savba.sk (P.O.J.); jaroslav.kovacik@savba.sk (J.K.); 2 IVMA STU, Vazovova 5, 812 43 Bratislava, Slovakia; stefan.emmer11@gmail.com; 3 Institute of Materials Science, Faculty of Materials Science and Technology in Trnava, Slovak University of Technology, Jána Bottu 25, 917 24 Trnava, Slovakia; roman.cicka@stuba.sk

**Keywords:** metal matrix composites, hot isostatic pressing, thermal expansion, Schapery model

## Abstract

Copper–graphite composites are promising functional materials exhibiting application potential in electrical equipment and heat exchangers, due to their lower expansion coefficient and high electrical and thermal conductivities. Here, copper–graphite composites with 10–90 vol. % graphite were prepared by hot isostatic pressing, and their microstructure and coefficient of thermal expansion (CTE) were experimentally examined. The CTE decreased with increasing graphite volume fraction, from 17.8 × 10^−6^ K^−1^ for HIPed pure copper to 4.9 × 10^−6^ K^−1^ for 90 vol. % graphite. In the HIPed pure copper, the presence of cuprous oxide was detected by SEM-EDS. In contrast, Cu–graphite composites contained only a very small amount of oxygen (OHN analysis). There was only one exception, the composite with 90 vol. % graphite contained around 1.8 wt. % water absorbed inside the structure. The internal stresses in the composites were released during the first heating cycle of the CTE measurement. The permanent prolongation and shape of CTE curves were strongly affected by composition. After the release of internal stresses, the CTE curves of composites did not change any further. Finally, the modified Schapery model, including anisotropy and the clustering of graphite, was used to model the dependence of CTE on graphite volume fraction. Modeling suggested that the clustering of graphite via van der Waals bonds (out of hexagonal plane) is the most critical parameter and significantly affects the microstructure and CTE of the Cu–graphite composites when more than 30 vol. % graphite is present.

## 1. Introduction

Electronic components in applications such as central processing units of computers, phones, broadcast radio and television receivers, and other daily used appliances in households and workplaces suffer from severe overheating. Silicon (Si) chips represent the typical electronic component that is usually produced. This chip, in commercial devices, needs to be maintained in a stress-free condition. [1,2,3]. For heat dissipation from the chip, the installation of a heat sink must be considered. The material of a heat sink should have high thermal conductivity. However, on the other hand, this material has to avoid any stress. Materials in contact with the chip should have a coefficient of thermal expansion (CTE) close to 4 × 10^−6^ K^−1^, that of Si [4,5].

Developing a high-performance heat sink for the purpose mentioned above has led to increasing industry requests for highly efficient materials. These are heat-dissipating materials with faster heat removal and minimization of thermal stresses [4,5]. More rapid heat removal can be achieved by higher thermal conductivity (TC), and thermal stresses can be reduced by modifying the coefficient of thermal expansion (CTE). Compatibility of searched materials with other materials in the electronics industry, in terms of thermal expansion, is also important [6]. 

Metals, especially copper, are the prime materials for heat sinks, due to their excellent thermal conductivity. However, their thermal expansion is almost eight times greater than the expansion of semiconductors. This mismatch causes thermal stress under operation, limiting the service life and efficient working conditions of high-power electronic devices [2]. Therefore, materials based on metal matrix composites (MMCs) are the most commonly developed for heat dissipation and show the highest potential for electronic applications. MMCs show several improvements, such as lower and tailorable CTE, high heat dissipation capability, low weight, and high dimensional stabilities at an elevated temperature. More specifically, there is great interest in MMCs based on Cu with a broad range of different reinforcements [3,7,8]. Since carbon (C) reinforcements have high thermal conductivity and low thermal expansion, MMCs reinforced with C can also present good TC and CTE [4]. In particular, copper matrix composites containing carbon in the form of carbon nanofibers (CNFs) [9,10] or carbon nanotubes (CNTs) [11] are considered promising as a future generation of these composite materials [12,13,14].

However, such composites have their disadvantages. The materials are still not cost-effective for broader commercialization, due to the high price of CNT, where the main challenge is to industrialize and transfer Cu/CNT from the lab bench to real-life use [15]. Compared with CNTs, graphene is easier to disperse into matrices and potentially more cost-effective [16,17]. Moreover, graphene has a larger surface area than CNTs, resulting in better transfer of its properties to the composite [18]. Therefore, graphene represents an alternative to CNTs in MMCs for structural and functional applications [19,20]. The lower coefficients of thermal expansion and density, combined with good thermal conductivity, render Cu–graphene composites ideal structural heat sink materials for microelectronic devices [21]. However, until now, Cu/graphene composites have had no commercial use. Graphite is another form of more cost-effective carbon used in available studies. Cu–graphite composites with graphite particles as embedded phases have been the focus of researchers for over three decades [2,22,23,24,25,26,27]. An important feature of these composites is almost no chemical reaction between the graphite and copper phases [28].

It is critically important to note that the final properties of composites depend not only on the fundamental constituents of the material but also on fabrication methods. Cu–C composite materials can be made in several ways. Some of them are: accumulative roll-bonding (ARB) [29,30], gas pressure infiltration of the molten Cu matrix into C powders, porous preform, or high modulus C fibers [31,32,33]. Another method is hot isostatic pressing (HIP) of the Cu and C powders [34]. The latter is also used in this study. The main objectives of this study are to investigate the dependence of the CTE of copper–graphite composites up to 90 vol. % graphite and to model the effective CTE of copper–graphite composites due to the anisotropy of the properties of the graphite phase. The modified Schapery model [35] that takes into account the anisotropy of the properties of the graphite phase and the clustering of graphite with increasing volume fraction of graphite will be used to validate the experimental measurements. 

## 2. Materials and Methods

Copper–graphite composites up to 90 vol. % graphite were prepared by wetly mixing pure copper powder with graphite powder in ethanol for 20 min in the Turbula type T2F device (WAB, Muttenz, Switzerland), followed by drying at 120 °C for 2 h in the universal oven Memmert UFP 400 (Memmert GmbH, Schwabach, Germany). Prior to consolidation, oxygen present at the surface of the copper powders was reduced under hydrogen in the electric tubular oven type 018LP (Elektrické pece Svoboda, Světice u Říčan, Czech Republic) at 400 °C for 2 h. The composites were then consolidated using hot isostatic pressing (HIPing) at 950 °C for 1.5 h under an argon pressure of 150 MPa (ASEA—Quintus QIH-6, Västerås, Sweden). Pure copper powder with particle size of <63 μm (average particle size of 22 μm, purity 99.9%), manufactured by Kovohuty Krompachy a.s., Slovakia, and graphite powder (average particle size of 16 μm, purity 99.9%) from Grafit a.s., Netolice, Czech Republic were used. The shape of the copper particles was dendritic, while graphite particles exhibited the flake shape. The sample with 90 vol. % graphite was investigated by X-ray nanotomography to assess the homogeneity of the copper spatial distribution in the graphite skeleton. X-ray nanotomography was performed employing the Phoenix/X-ray Nanotom 180 (Waygate Technologies, Hürth, Germany). Microstructural and chemical analyses were performed by a JEOL 6610 scanning electron microscope (JEOL, Tokyo, Japan) equipped with an energy dispersive X-ray analyzer, OI X-max 50 mm^2^ (Oxford Instruments, Abingdon, UK). A NETZSCH 402C dilatometer (Netzsch, Selb, Germany) was used for monitoring thermal expansion. The sample temperature was measured by an S-type thermocouple located in close proximity to the sample. The samples were heated from 30 °C to 380 °C at a heating rate of 3 °C/min in an inert argon atmosphere. All measurements were repeated two or three times to obtain a dataset for statistical analysis. The correction measurements were performed using alumina standards under the same experimental conditions. The linear CTE was calculated as defined by the equation:

(1) $$\alpha =\varepsilon \frac{1}{\Delta T}=\frac{\Delta l}{l_{0}}\frac{1}{\Delta T^{\prime }}$$

where α is the coefficient of thermal expansion; Δ*T′* = *T* − *T*_0_ is the temperature interval (*T*_0_ = 293 K); *l*_0_ is the length of the sample before testing; Δ*l* is the expansion for Δ*T*: Δ*l* = l − *l*_0_; and *ε* = Δ*l*/*l*_0_ is the relative length change. The calibration and investigated sample measurements were performed according to DIN 51045 with NBS-Pt. The CTE values at room temperature were estimated from a linear regression of the experimentally obtained dependence of CTE on temperature. The CTE values derived for temperature from 100 °C to 380 °C were only considered for linear fitting.

Tested samples had the shape of a disc with dimensions ø 5 mm × 15.0 mm. Top and bottom surfaces were machined with a tolerance of ± 0.02 mm. The sample density was calculated from the known geometry and weight. For each graphite amount, three different samples were prepared. Average values were used to analyze the importance of the value of CTE. 

O and H concentrations were measured with a GFA Bruker G8 Galileo device (Bruker, Karlsruhe, Germany) following the bulk and powdered samples melt extraction method, based on calibrated O and H known ppm concentration curves. Melting the samples releases oxygen and hydrogen, if present. O reacts with carbon from the graphite crucible, forms monoxide (CO), travels with the gas stream (He), and is detected and measured with a dual-range CO infrared detector. H-containing gas stream carrier (N_2_) passes through a thermal conductivity cell (TCD), where ppm concentration corresponds to the gas mix’s conductivity difference with pure carrier gas. We collected three measurements for each sample.

## 3. Results and Discussion

### 3.1. Microstructural and Chemical Analysis Using SEM-EDS Microscopy

**Figure 1:**
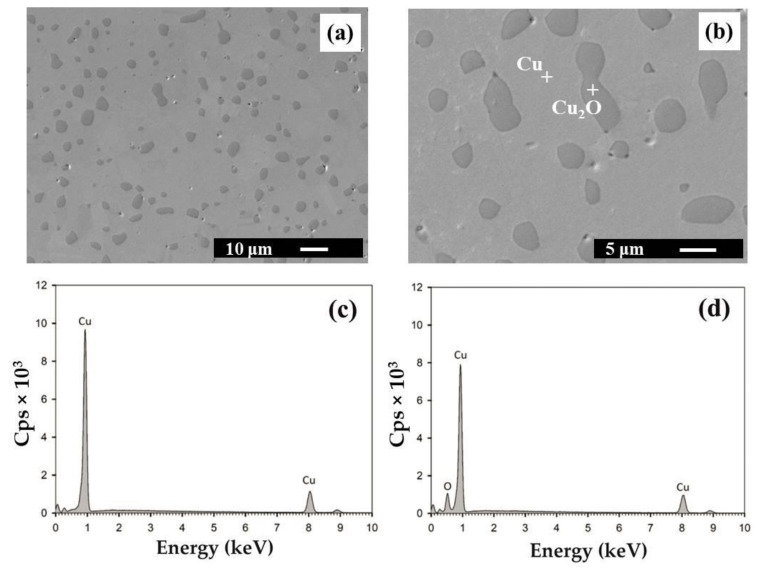
SEM microstructure of the HIPed Cu sample at magnification 1000× (**a**) and 3000× (**b**). Cu and Cu_2_O are colored in light and dark grey, respectively. EDS spectra were obtained by point analysis in Cu (**c**) and Cu_2_O (**d**).

**Figure 2:**
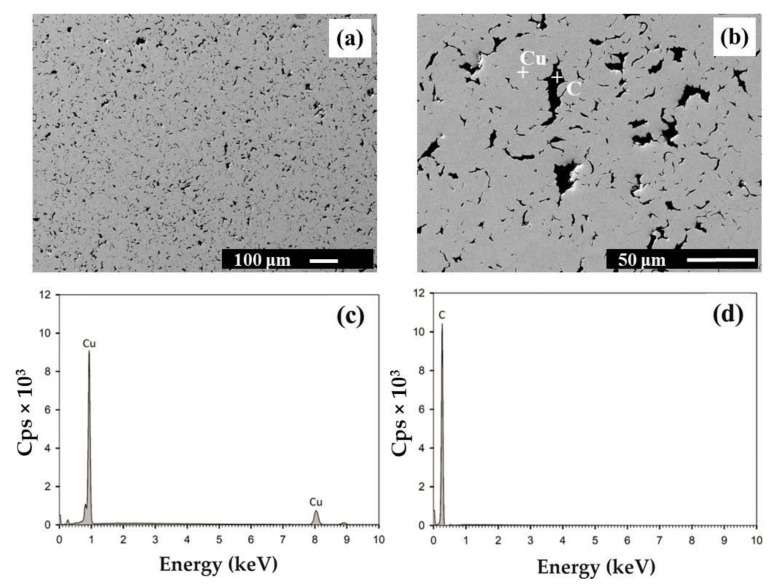
SEM microstructure of the Cu–graphite sample with 10 vol. % graphite at magnification 100× (**a**) and 500× (**b**). Cu and graphite are colored in light grey and black, respectively. EDS spectra were obtained by point analysis in Cu (**c**) and C (**d**).

**Figure 3:**
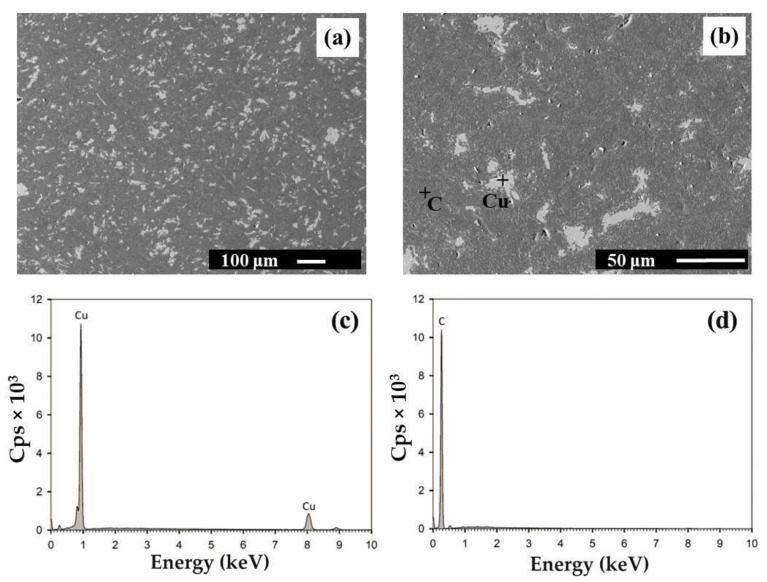
SEM microstructure of the Cu–graphite sample with 90 vol. % graphite at magnification 100× (**a**) and 500× (**b**). Cu and graphite are colored in light and dark grey, respectively. EDS spectra were obtained by point analysis in Cu (**c**) and C (**d**).

The microstructure of HIPed Cu (Figure 1) and Cu–graphite composites with 10 and 90 vol. % was investigated by SEM equipped with an EDS analyzer. SEM investigations showed that the copper and graphite phases were homogeneously dispersed in tested composites (Figure 2 and Figure 3). With changing volume fraction of graphite, a percolation phase transition occurred in the graphite phase. Particularly, isolated graphite clusters slowly started to become interconnected. At a certain graphite volume fraction, the graphite skeleton inside the copper skeleton structure began to occur. Finally, the graphite phase tended to break up the copper skeleton into isolated clusters. However, as demonstrated further by X-ray tomography, even for 90 vol. % graphite, a very thin 3D copper skeleton still existed inside the graphite skeleton.

### 3.2. Microstructural Analysis Using X-ray Tomography

The sample with 90 vol. % graphite was investigated using X-ray nanotomography to better understand the spatial distribution of the copper phase. Samples with a low volume fraction of graphite were not suitable for the investigation, because copper absorbs X-rays very well. Thus, it was impossible to obtain a sharp 3D object or gain any meaningful information from the generated 3D object.

**Figure 4:**
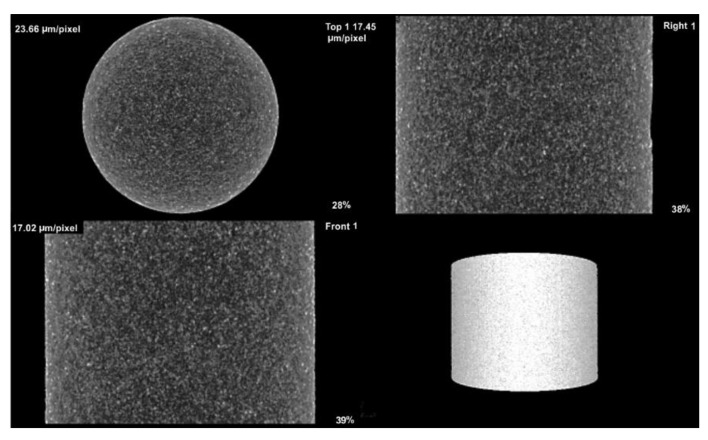
The Cu–graphite sample with 90 vol. % graphite investigated using X-ray tomography. Graphite is in dark grey (sample diameter is 8 mm).

**Figure 5:**
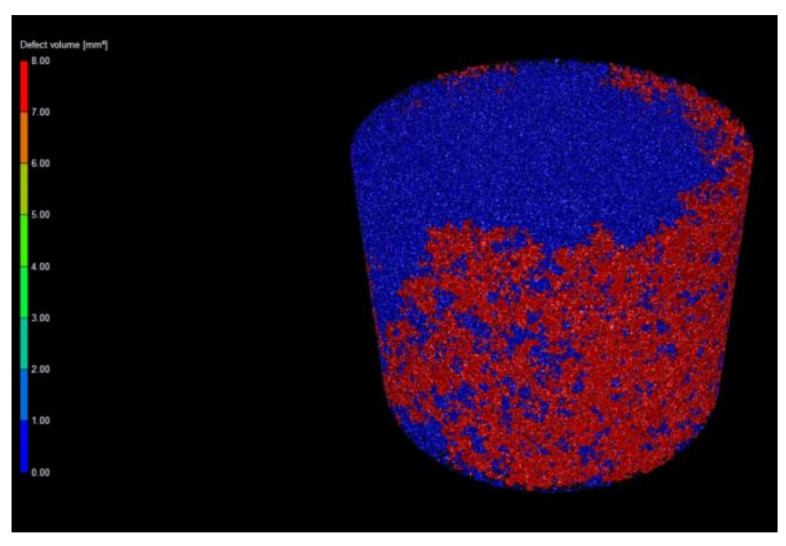
The Cu–graphite sample with 90 vol. % graphite investigated using X-ray tomography: a surface cluster of copper is in red; volumetric copper cluster is in blue (sample diameter is 8 mm).

X-ray tomography confirmed the SEM results: the sample containing 90 vol. % graphite contained a homogeneously dispersed copper phase in the entire volume of the composite (Figure 4). Figure 5 also indicates that, according to 3D images, a continuous copper skeleton was built inside the continuous graphite skeleton (volumetric copper cluster at Figure 5), similar to carbon–copper composites produced by gas pressure infiltration technology [32]. This microstructure likely significantly affects the values of CTE and other physical and mechanical properties of the Cu–graphite composites with a high volume fraction of graphite.

### 3.3. Thermal Properties

**Figure 6:**
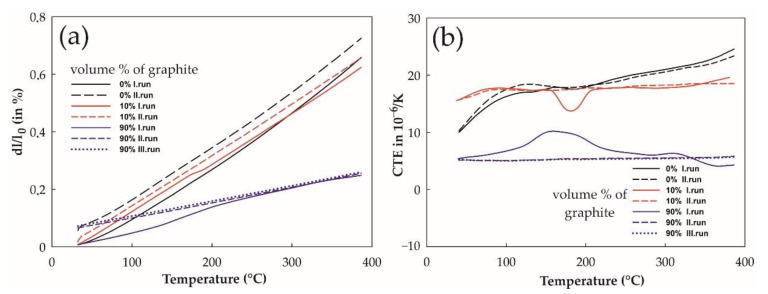
Dependence of relative elongation (**a**) and coefficient of thermal expansion (**b**) on temperature for the Cu HIPed (black), Cu + 10% graphite (red), and Cu + 90% graphite (blue) samples. The first run (solid line), the second run (short dash line), and the third run (dotted line) are displayed.

Heating the copper sample from 30 to 380 °C resulted in permanent prolongation by 0.06% after the first dilation run (Figure 6). This could be attributed to the presence of Cu_2_O of micrometer size in the copper structure, due to the presence of oxide on the surface of the copper particles before HIPing [36]. Comparison of the CTE values from the first and the second runs pointed to the effective release of residual tensions during the first heating. 

For composite Cu + 10 vol. % graphite, the elongation was linear up to about 170 °C in the first cycle, and then the elongation changed slope to about 193 °C, then went up the same slope. This finding is also reflected in the transient decrease in the CTE value (Figure 6b) with minima around 183 °C. After the first cycle, the sample remained elongated by about 0.02%. This could be connected with the release of internal stresses locked around the graphite phase, due to the HIPing technology of preparation. The second cycle was already linear over the entire heating range.

The expansion of the Cu + 90 vol. % graphite sample is mostly governed by expansion of graphite, with the significantly lower CTE (4.3 × 10^−6^ K^−1^) in comparison to pure copper (18.45 × 10^−6^ K^−1^). Because the CTE of the Cu + 10 vol. % graphite composite is predominantly controlled by the expansion of copper, the decrease in the CTE value with increasing graphite volume fraction was expected.

The first heating run of the Cu + 90 vol. % graphite sample caused the highest permanent prolongation of 0.06%. The higher values with increasing graphite phase size are due to clustering of graphite content in the prepared composite. Moreover, the elongation graph changed slope between 134 and 205 °C. CTE is nonlinear in this case, with maximum at 165 °C and minimum at 363 °C. This could be again connected with the release of internal stresses locked around the dominating graphite phase, due to the HIPing technology of preparation. Notably, the second heating was not accompanied by changes in the thermal behavior of the sample. This was also confirmed by the third run, which did not induce any additional changes. Both have CTE values at the end of cycle of 5.86 × 10^−6^ K^−1^ and 5.8 × 10^−6^ K^−1^, respectively.

To summarize the internal stresses in prepared composites are released during the first heating cycle of measuring the coefficient of thermal expansion. The composition affects permanent prolongation after the first run and the shape of measured elongation and CTE curves.

### 3.4. Modelling of CTE Dependence on the Composition

**Figure 7:**
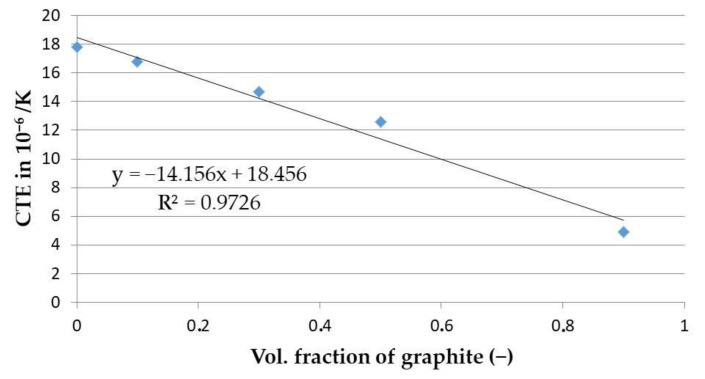
The CTE values of the Cu–graphite composites fitted by a straight line.

The CTE values of the composites were extrapolated down to RT using measured data to improve RT data precision. It was observed that at room temperature, the coefficient of thermal expansion of Cu–graphite composites decreased with increasing graphite volume fraction from 17.8 × 10^−6^ K^−1^ for the HIPed pure copper sample to 4.9 × 10^−6^ K^−1^ for the 90 vol. % graphite. The observed dependence on graphite volume fraction can be modeled using linear regression; dependence of the Cu–graphite composites’ coefficients of thermal expansion on the volume fraction of graphite was fitted using the least square method: α_comp_ = (−14.15.n_Vgr_ + 18.45) × 10^−6^ K^−1^ with an R^2^ value of 0.972. From this approach (see Figure 7), the estimated average CTE value of pure copper is 18.45 × 10^−6^ K^−1^, and the average value of graphite is 4.3 × 10^−6^ K^−1^.

**Figure 8:**
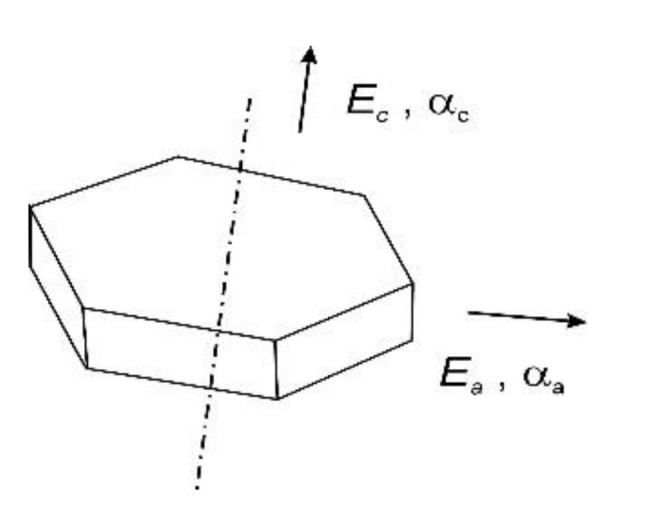
Definition of anisotropy of the graphite powder used in the model: *a*—perpendicular to the central axis; *c*—parallel to the central axis [37].

The resulting values of the coefficients of thermal expansion of the composites are affected by the composition, anisotropy of graphite, and clustering of the graphite phase due to the preparation method. To describe these effects, a simple model by Schapery [35] was chosen for CTE prediction:

(2) $$\alpha =\frac{\alpha_{a}E_{a}V_{a}+\alpha_{c}E_{c}V_{c}\;+\alpha_{Cu}E_{Cu}V_{Cu}}{E_{a}V_{a}+E_{c}V_{c}+E_{Cu}V_{Cu}}$$

where *α*, *V*, and *E* are the CTE, volume fraction, and Young’s modulus, respectively. The properties of graphite in the perpendicular and parallel direction to the main powder axis are represented by *a* and *c* (Figure 8). Cu subscripts denote the properties of the copper.

**Figure 9:**
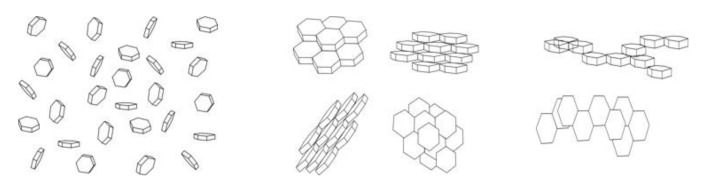
Sketch of various possible fillings of the copper matrix with graphite: left—entirely separated; center—clustered predominantly in *c*-direction; right—clustered predominantly via *a*-direction [37].

The raw materials’ properties are practically identical; the main difference is in the interface area. In the case of graphite, powder clustering occurs, i.e., the graphite powders are more or less packed together. The higher the degree of clustering, the lower is the interface area. Due to the anisotropy of graphite properties (positive and negative CTE), different clustering (Figure 9) can produce different CTEs of composites. 

The overall volume of graphite can be divided into two parts, one corresponding to the copper–graphite interface with dominant positive CTE of graphite and the second one with dominant negative CTE of graphite. It can be assumed that *V_a_* = *c_a_*·*V_G_* and *V_c_* = *c_c·_V_G_*, where *c*_a_ and *c*_c_ represent the fraction of graphite volume *V_G_* acting in the resulting composite with negative and positive CTE, respectively. It is notable that *c_a_* + *c_c_* = 1, because *V_a_* + *V_c_* = *V_G_*. Using these presumptions, the Schapery model changes to:

(3) $$\alpha =\frac{\left(\alpha_{a}E_{a}c_{a}+\alpha_{c}E_{c}\left(1-c_{a}\right)\right)\;\cdot V_{G}+\alpha_{Cu}E_{Cu}V_{Cu}}{\left(E_{a}c_{a}+E_{c}\left(1-c_{a}\right)\right)\;\cdot V_{G}+E_{Cu}V_{Cu}}$$

Then, the proposed model was used to calculate the CTE of composites at room temperature (see Table 1). The value of *c_a_* was varied to obtain the identical value of the composite CTE as measured. Graphite CTE values were used according to [38]. Values of the mechanical properties of graphite were roughly assessed according to the study of Koráb et al. [16]. 

For the studied composites at low graphite concentration, the CTE values are predominantly governed by graphite properties themselves, predominantly in the direction of hexagonal graphite structure (c_a_ = 0.55 at 10 vol. % graphite). The result is similar to the value of *c_a_* ≈ 0.51 in coated Cu–graphite samples [37]. Note: the *c_a_* values for coated samples were recalculated using a newly obtained CTE pure copper value of 18.45 × 10^−6^ K^−1^.

**Figure 10:**
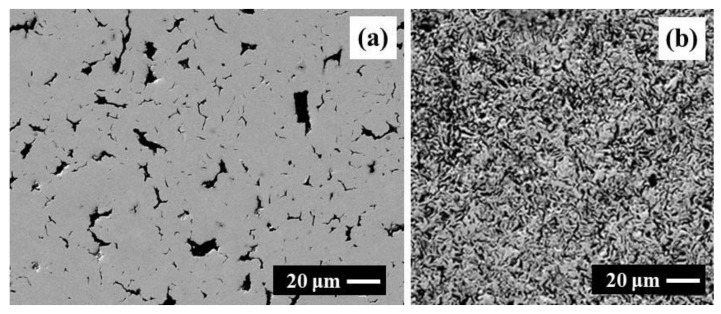
SEM images of the Cu–graphite composites. Cu are colored in light grey, and graphite is colored in black: (**a**)—uncoated graphite at 10 vol. % graphite; (**b**) [37]—coated graphite at 50 vol. % graphite.

This indicates that the proposed model probably works well. At low graphite concentrations, the graphite phase is homogeneously distributed. The graphite phase is also dispersed in the copper matrix without clustering of graphite. This microstructural feature coincides qualitatively with the composite microstructure at 50 vol. % graphite when coated graphite is used (Figure 10).

At higher graphite concentrations, the clustering of graphite powders via van der Waals bonds (out of hexagonal plane) increases. Table 1 indicates that it takes place very early, even below 30 vol. % graphite (*c_a_* = 0.39). After this threshold, the *c_a_* value decreased and seemed to be independent of composition; at 90 vol. % graphite, the *c_a_* value is 0.38. For the average CTE value of graphite, the estimated *c_a_* value is 0.29.

### 3.5. Chemical OHN Analysis

SEM detected cuprous oxide (Cu_2_O) in the microstructure of the pure HIPed copper sample. This finding was also proved by OHN analysis. The measured oxygen content was 5062 ± 1128 ppm of O (Table 2). This amount corresponds to 0.5 wt. % of oxygen in the pure HIPed copper sample. Evidently, this is due to copper powder oxidation; it starts to react with oxygen after exposure to the surrounding atmosphere. Copper oxides exist in two different forms: cupric oxide (CuO) and cuprous oxide (Cu_2_O), depending on the valence state of copper [39]. Oxidation of copper has two oxide layers, namely Cu_2_O, which is on the surface of copper and is present at all temperatures, and CuO, which is on the Cu_2_O layer in the temperature range of 250 to 1030 °C and dissociates above 1030 °C [40]. Due to the reduction process, only traces of Cu_2_O were observed in the HIPed copper sample.

Cu_2_O was not detected by SEM in the composite microstructures (Cu + 10 vol. % – Cu + 90 vol. %). This finding was also proved by OHN analyses: at 10 vol. % and 50 vol. % graphite, the oxygen content was 1027 ± 202 ppm of O and 2262 ± 292 ppm of O, respectively. These values are comparable to the oxygen content in the original graphite powder, 2007 ± 321 ppm of O. This is partially due to the presence of graphite powder, which enables better access of hydrogen during the reduction of powder mixtures at 400 °C prior to consolidation. 

At 90 vol. % graphite, a very high amount of oxygen was observed, 16,006 ± 810 ppm of O (Table 2). Nevertheless, only for this graphite content was a high amount of hydrogen also detected, 2187 ± 8 ppm of H. From this, the ratio of oxygen to hydrogen is 7.32. It is comparable to the ratio of oxygen’s atomic mass to the atomic mass of the two hydrogen atoms in water (7.936). This is probably due to water adsorption inside the graphite skeleton on the surfaces of stacked graphite powders (humidity in atmosphere). At 90 vol. %, the graphite phase is fully exposed to the surrounding environment. 

90 vol. % graphite is probably most prone to water adsorption on the surface of the graphite skeleton consisting of stacked graphite powders, where nanometer pores can exist in such composite material. This intercalated water [41] inside the graphite structure probably originated from the humidity in the atmosphere. 

In summary, OHN analyses confirmed Cu_2_O oxide observed by SEM in the microstructure of pure HIPed copper. At least 0.5 wt. % oxygen was present. Significantly lower oxygen concentrations in 10 and 50 vol. % graphite indicated that, in the presence of graphite, the copper oxide was reduced considerably during the preparation method. At 90 vol. % graphite, a very high amount of oxygen was observed in the composite sample, probably due to absorption of water from atmospheric humidity after the preparation of samples.

## 4. Conclusions

Cu–graphite composites with 10 and 90 vol. % graphite were prepared from the mixture of copper and graphite powders by hot isostatic pressing (HIPing) to investigate the coefficient of thermal expansion over the whole range of graphite content, including the pure HIPed copper sample. The coefficient of thermal expansion decreased from 17.8 × 10^−6^ K^−1^ for HIPed pure copper to 4.9 × 10^−6^ K^−1^ for 90 vol. % graphite. The observed dependence on the volume fraction of graphite (n_Vgr_ ) is almost linear. It was fitted to a line by the least square method: (−14.15.n_Vgr_ + 18.45) × 10^−6^ K^−1^ with R^2^ = 0.972. The estimated average CTE value of graphite is 4.3 × 10^−6^ K^−1^.

It was observed that the internal stresses in the composites were released during the first heating of the CTE measurement. The permanent prolongation after the first run and the shape of elongation curves and CTE curves are strongly affected by increasing graphite volume fraction. Maximal permanent prolongation was 0.06% for 90 vol. % graphite. This needs to be taken into account if industrial applications require high stability of geometry. Finally, after the release of internal stresses, the elongation and CTE curves of the second and third runs did not change any further.

The modified Schapery model, considering the anisotropy of graphite, was used to predict the experimentally observed CTE values. It enabled modeling the structural changes in composite structure due to the clustering of graphite particles during composite preparation. It was obtained from the model that, at low graphite content, the clustering of graphite is negligible (*c_a_* = 0.55 at 10 vol. % graphite). The value is in good agreement with the *c_a_* = 0.5 for 50 vol. % graphite when copper-coated graphite was used [37] (the same graphite powder as in the present work), and coating prevented the clustering of the graphite phase. Clustering via van der Waals bonds (out of hexagonal plane) started to occur with increasing graphite volume fraction at 30 vol. % graphite (*c_a_* = 0.39). Then, besides robust clustering, the *c_a_* is almost constant, and at 90 vol. % graphite, it was *c_a_* = 0.38.

The obtained results indicate that even a simple model, after considering the anisotropy of material properties (if model assumptions enable it), can show significant structural changes in the composite materials with composition. This information can be beneficial for further micromechanical modeling [24] of composite materials and precise determination of the cross-property [27] relations for these materials.

The obtained results indicate that prepared composites with high volume fractions of graphite have a coefficient of thermal expansion approaching the CTE of Si. In any case, further research is required in the future.

## Figures and Tables

**Table 1:** Measured CTE, model parameters, and calculated CTE for the investigated Cu–graphite composites at room temperature.

	α_meas._	α_a_	α_c_	α_Cu_	*E*_a_	*E*_c_	*E*_Cu_	*c*_a_	α_calc._
	(10^−6^ K^−1^)	(10^−6^ K^−1^)	(GPa)	(GPa)	(GPa)	(-)	(10^−6^ K^−1^)	(-)	(-)
HIP Cu	17.8	0	0	18.45	0	0	125		17.8
Cu + 10%	16.8	−1	26	18.45	200	20	125	0.550	16.8
Cu + 30%	14.7	−1	26	18.45	200	20	125	0.390	14.7
Cu + 50%	12.6	−1	26	18.45	200	20	125	0.335	12.6
Cu + 90%	4.9	−1	26	18.45	200	20	125	0.388	4.9

**Table 2:** The results of OHN analyses for the tested Cu–graphite composites, pure HIPed copper, and original graphite powder.

Composition	O (ppm)	H (ppm)	O/H
HIP Cu	5062 ± 1128	-	-
Cu + 10 vol. % graphite	1027 ± 202	38 ± 5	27.03
Cu + 50 vol. % graphite	2262 ± 292	196 ± 2	11.54
Cu + 90 vol. % graphite	16,006 ± 810	2187 ± 4	7.32
Graphite powder	2007 ± 321	170 ± 8	11.81

## Data Availability

The data presented in this study are available on request from the corresponding author. The data are not publicly available due to funding agency.
